# Phylogenetic Responses of Marine Free-Living Bacterial Community to *Phaeocystis globosa* Bloom in Beibu Gulf, China

**DOI:** 10.3389/fmicb.2020.01624

**Published:** 2020-07-16

**Authors:** Nan Li, Huaxian Zhao, Gonglingxia Jiang, Qiangsheng Xu, Jinli Tang, Xiaoli Li, Jiemei Wen, Huimin Liu, Chaowu Tang, Ke Dong, Zhenjun Kang

**Affiliations:** ^1^Key Laboratory of Environment Change and Resources Use in Beibu Gulf, Ministry of Education, Nanning Normal University, Nanning, China; ^2^Department of Biological Sciences, Kyonggi University, Suwon-si, South Korea; ^3^Guangxi Key Laboratory of Marine Disaster in the Beibu Gulf, Beibu Gulf University, Qinzhou, China

**Keywords:** *Phaeocystis globosa*, phylogenetic relatedness, 16S rRNA, harmful algal blooms, Beibu Gulf, South China Sea

## Abstract

*Phaeocystis globosa* blooms are recognized as playing an essential role in shaping the structure of the marine community and its functions in marine ecosystems. In this study, we observed variation in the alpha diversity and composition of marine free-living bacteria during *P. globosa* blooms and identified key microbial community assembly patterns during the blooms. The results showed that the Shannon index was higher before the blooming of *P. globosa* in the subtropical bay. *Marinobacterium* (γ-proteobacteria), *Erythrobacter* (α-proteobacteria), and *Persicobacter* (Cytophagales) were defined as the most important genera, and they were more correlated with environmental factors at the terminal stage of *P. globosa* blooms. Furthermore, different community assembly processes were observed. Both the mean nearest relatedness index (NRI) and nearest taxon index (NTI) revealed the dominance of deterministic factors in the non-blooming and blooming periods of *P. globosa*, while the bacterial communities in marine waters after the blooms tended to be controlled by stochastic factors. Our findings revealed that the assembly of the bacterial community in marine *P. globosa* blooms is a complex process with mixture effects of marine microbiomes and environmental parameters.

## Introduction

The marine phytoplankton community is a main contributor to primary production and plays important roles in regulating energy export, carrying out more than half of the photosynthetic carbon fixation in euphotic zones (Keller and Riebesell, 1989; Hopcroft and Roff, 2003; Reusch and Boyd, 2013; Bernd et al., 2015; Tamm et al., 2018), and contributing substantially to global climate regulation (Keller and Riebesell, 1989; Reusch and Boyd, 2013). The assembly of certain phytoplankton communities appears to be activated or accelerated by physical, chemical, and biological factors in marine ecosystems (Rilov and Treves, 2010; Reusch and Boyd, 2013; Sunagawa et al., 2015). The phytoplankton genus *Phaeocystis* (Prymnesiophyceae), which contains at least six species, is globally distributed and often dominates the phytoplankton communities in temperate and polar oceans (Elzenga et al., 2000; Brussaard et al., 2004; Schoemann et al., 2005; Delmont et al., 2014; Lars et al., 2017). In marine ecosystems, the blooms of *P. globosa* colonies are recognized as harmful algal blooms (HABs) (Schoemann et al., 2005; Wang et al., 2010; Zhang et al., 2017). The life-cycle transformation caused by abiotic or biotic stress on *P. globosa* changes local ecosystem functions ranging from nutrient utilization and trophic energy transfer to the export of allelochemical compounds and production of dimethyl sulfide (DMS, an important climate-cooling aerosol) to restructure microbial food webs (Dutz and Koski, 2006; Reigstad and Wassmann, 2007). Evidence supports that during blooms, *P. globosa* utilizes the colony skin as defenses against predation and attack from other organisms, especially parasitic microbes (Hamm et al., 1999; Dutz and Koski, 2006; Verity et al., 2007). However, there is little information regarding the changes in free-living bacterial composition and bioindicators during natural *P. globosa* blooms.

From the viewpoint of ecological processes, microbial communities are driven by a combination of stochastic and deterministic processes (Stegen et al., 2012; Wang et al., 2013; Espinosaasuar et al., 2015). Deterministic forces include biotic interactions, environmental conditions, and species sorting, while stochastic processes include random birth–death cases and ecological drift (Chase and Myers, 2011; Stegen et al., 2012). A combination of both sets of processes might cause phylogenetic clustering or overdispersion of community composition (Horner-Devine and Bohannan, 2006; Wang et al., 2013; Singer et al., 2018). Previous studies suggested that marine the microbial community structure differs substantially among *P. globosa* blooms, with changes in nutrient concentrations and other environmental factors (Wang et al., 2010; Bernd et al., 2015). These patterns suggest a dominant role of *P. globosa* blooms in microbial community assembly. However, the phylogenetic responses and ecological processes driving marine microbial community assembly among *P. globosa* blooms are still poorly understood.

For decades, blooms of *P. globosa* have occurred frequently in coastal regions of southern China during both winter and summer, greatly impacting local fishing, farming, and environmental health (Wang et al., 2006, 2010). Recently, *P. globosa* blooms have occurred frequently along the Beibu Gulf coast (Liu et al., 2015; Qin et al., 2016; Xu et al., 2017), a historically important region for the economies of China and Vietnam because of the abundant and diverse marine resources (Zheng et al., 2012). As such, there is a need to understand the impact of algal blooms and the mechanisms of their impact on ecosystem health. In this study, we investigated the phylogenetic response of the marine free-living bacterial community to a harmful *P. globosa* bloom. We aimed to answer the following questions: (i) How do environmental factors influence prokaryotic community structure and diversity during *P. globosa* blooms? (ii) How does *P. globosa* affect ecological processes (deterministic or stochastic) driving the response of the marine bacterial community to *P. globosa* blooms? (iii) Which are the key prokaryotic species associated with *P. globosa* blooms in the local ecosystem?

## Materials and Methods

### Study Sites and Sampling Protocols

The surface seawater samples used in this study were collected from a water depth of 0.5 m using a rosette of Niskin bottles attached to a CTD probe frame during the Open Cruises of Qinzhou Bay (Supplementary Figure S1) on November 11th (2017, before *P. globosa* blooms, BB group), December 10th (2017, during *P. globosa* blooms, DB group), and February 6th (2018, after *P. globosa* bloom, AB group). In each stage, triplicate water samples were collected from eight stations. A total of 24 surface water samples were analyzed in this work. The water temperature, pH, salinity, and dissolved oxygen (DO) concentration of each subsample were measured using a portable meter (556 MPS; YSI, United States). Following collection, samples were stored on ice for transport to the laboratory. For DNA extraction, five liters of surface seawater was filtered sequentially through 0.8 and 0.45-μm filters (Nalgene, Rochester, NY, United States) to remove debris and larger organisms, then filtered through a 0.22-μm Millipore filter to target only the free-leaving bacterial community, and the 0.22-μm filters were stored at -20°C for subsequent DNA analysis. The specific sampling sites and nutrients are listed in Supplementary Table S1.

### Nutrient Analysis

One-liter seawater samples were collected in parallel, filtered through filters (47 mm, Whatman GF/F nominal pore size, Maidstone, United Kingdom), and frozen at −20°C until analysis. The concentrations of total dissolved nitrogen (TDN), NO_3_^–^, and NH_4_^+^ were determined using the Cu–Cd column reduction and indophenol blue color formation methods, respectively (Science et al., 1999). The analysis of NO_2_^–^ was based on the reaction of an aromatic amine with NO_2_^–^; the reaction product was quantified using spectrophotometric methods (Science et al., 1999). PO_4_^3–^ was measured by a modified single-solution method (Murphy and Riley, 2014). The amount of total organic carbon (TOC) was measured using the standard methods (Clescerl, 1998). Chl *a* on the filter (47 mm, GF/F membranes, Whatman) from 500 ml of seawater was extracted using 10 ml of 90% (v/v) acetone at 4°C in the dark for 24 h, and the extractions were analyzed using a fluorescence spectrophotometer (F-4500, Hitachi Co., Tokyo, Japan) after centrifugation, according to the procedure of a previous study (Parsons et al., 1984). After the frozen samples were thawed, SiO_3_^2–^ concentrations were determined by a nutrient analyzer (Lachat Quickchem 8500, United States) according to the standard manual (Zhou et al., 2015).

### Number and Size of *P. globosa*

To study *P. globosa* colonies before disintegration by fixation and sonification, 10-ml aliquots were screened for colony appearance on an inverted microscope. The number of colonies was counted immediately when samples arrived at the laboratory. *P. globosa* colony size measurements were made with a Zeiss and an Olympus Vanox microscope with a 40× objective lens (eyepiece graticule units: 2.5 μm). The number of free-living *P. globosa* cells was estimated based on the regression relationship between colony diameter and cell number per colony (LogY = 1.349 logX - 0.44, where Y is the cell number in the colony and X is the diameter of the colony, Huang et al., 2012).

### DNA Extraction, PCR, and Pyrosequencing

Total DNA of the 24 samples was extracted (Mo-Bio PowerWater DNA Isolation Kit, Carlsbad, CA, United States) from the 0.22-μm filters. DNA yield and purity were measured by a NanoDrop spectrophotometer (Delaware, United States). For sequencing, the V3 and V4 regions of the 16S ribosomal RNA gene from the seawater were amplified with the bacterial primers 341F (CCTACGGGNGGCWGCAG) and 805R (GACTACHVGGGTATCTAATCC) with sample-specific barcodes (Klindworth et al., 2013). Two microliters of template DNA was aliquoted into illustra^TM^ PuReTaq Ready-To-Go^TM^ PCR Beads (GE Healthcare, Waukesha, WI, United States) with 20 μL of PCR-grade molecular water and 1.5 μL of each primer for a total reaction mixture of 25 μL. PCR was conducted on a Bio-Rad thermocycler (Hercules, CA, United States), and the conditions were as follows: 1 min of initial denaturation at 95°C, 35 cycles of 30 s of denaturation at 95°C, 30 s of annealing at 52°C, 1 min of elongation at 72°C, and a final extension for 10 min at 72°C. The PCR products were confirmed with 2% agarose gel electrophoresis. Throughout the DNA extraction process, ultrapure water, instead of a sample solution, was used to exclude the possibility of false-positive PCR results as a negative control. Three technical replicates of the PCR were conducted for each sample and prepared for MiSeq sequencing. Finally, 72 PCR products were purified by AMPure XT Beads (Beckman Coulter Genomics, Danvers, MA, United States). The size and quantity of the amplicon library were assessed on an Agilent 2100 Bioanalyzer (Agilent, United States) and with the Library Quantification Kit for Illumina (Kapa Biosciences, Woburn, MA, United States). The purified library was diluted, denatured, and rediluted; mixed with the PhiX (expected at 30%) control library (v3); constructed using a Nextera XT sample preparation kit (Illumina) according the manufacturer’s instructions; and then submitted to a 250PE MiSeq system for sequencing with the standard Illumina sequencing primers with a 12-mer barcode sequence for 600 cycles. Raw data files in FASTQ format were deposited in the NCBI Sequence Read Archive (SRA) under BioProject number PRJNA565408.

### Taxonomic Analysis

Sequencing data were analyzed using the single software platform MOTHUR v.1.35.1 (Schloss et al., 2009). Denoising included the removal of sequences with primer mismatches or a length >275 bp and screening for sequences with quality scores <30 (with the MOTHUR commands qwindowaverage = 30 and qwindowsize = 100; screen.seqs maxambig = 0, maxlength = 275). According to the Schloss SOP pipeline^1^, sequences were trimmed to a length of 250 bp, screened for chimeras (chiumera.vsearch dereplicate = t), and grouped into OTUs (dist.seqs, cutoff = 0.03) (Edgar et al., 2011; Schloss and Westcott, 2011). After the removal of barcodes and primers, the remaining sequences were trimmed so that all sequences that started and ended at similar positions in their alignment to the SILVA database (Edgar et al., 2011) were removed. After the removal of any plastid and non-16S rRNA bacterial or archaeal reads, sequences were classified using the Ribosomal Database Project (RDP) Naïve Bayesian Classifier (minimum confidence of 50%) (Wang et al., 2007).

### Phylogenetic Structure Analysis

Phylogenetic maximum likelihood-approximation trees were constructed in FastTree software (Price et al., 2010). Rare OTUs with fewer than five reads per sample were excluded to reduce sequencing bias. To quantify the phylogenetic structure metric within each sample, we calculated the nearest relatedness index (NRI) and the nearest taxon index (NTI) across all samples using mntd, ses.mntd, and condistnt in the package picante of R (Kembel et al., 2010; Stegen et al., 2012).

For a single community, an NRI and NTI greater than +2 indicate that coexisting taxa are more closely related than expected by chance (phylogenetic clustering), indicating a dominant role of deterministic environmental filtering (Horner-Devine and Bohannan, 2006; Stegen et al., 2012; Zhou and Ning, 2017). An NRI and NTI less than -2 indicate that coexisting taxa are more distantly related than expected by chance (phylogenetic overdispersion), indicating a dominant role of negative interactions (e.g., competition) (Horner-Devine and Bohannan, 2006; Stegen et al., 2012; Zhou and Ning, 2017). Meanwhile, -2 < mean NTI or mean NRI < +2 means that stochastic or ecologically neutral factors play an important role in community assembly (Zhou and Ning, 2017).

### Statistical Analysis

Data normality was tested using the Shapiro–Wilk test. After filtering of the raw OTU table, the diversity within each bacterial community (α-diversity) was assessed by diversity indices, including the Shannon index, the Chao index (SChao), the Simpson index (1/D), and Good’s coverage. Additionally, ANOVA, PERMANOVA, and Spearman’s correlation were performed using the RAM and vegan packages in the R language. Random forest analysis was applied to obtain the important indicator taxa using the random forest package with 1000 trees and default settings (Au, 2017). A linear regression of the ln-transformed data of the Bray–Curtis distances plotted against the ln-transformed environmental distances was used to estimate the relationship between environmental heterogeneity and bacterial community (Wang et al., 2017). Redundancy analysis (RDA) of the Hellinger-transformed data was also run in R, followed by ANOVA with 999 permutations. Variation partitioning analysis (VPA) was conducted to examine the contribution of environmental factors to the microbial communities according to the RDA. Partial least-squares path modeling (PLS-PM) was selected to analyze the effects of various environmental factors on microbial activities. PLS-PM was conducted using the plspm package (Kembel et al., 2010). The NTI was used to characterize the phylogenetic relatedness within each community, and the Shannon index was selected to indicate microbial diversity within each sample. All significant differences were defined at *p* < 0.05 or *p* < 0.01.

## Results

### Variation in the Alpha Diversity and Community Composition of Marine Bacteria During *P. globosa* Blooms

**FIGURE 1:**
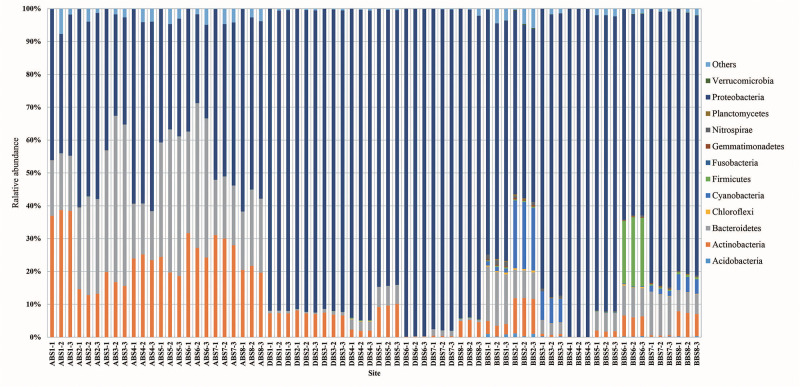
The relative abundances of bacterial communities at the phylum level during the *P. globosa* blooms. BB, before *P. globosa* blooms; DB, during *P. globosa* blooms; AB, after *P. globosa* blooms.

**FIGURE 2:**
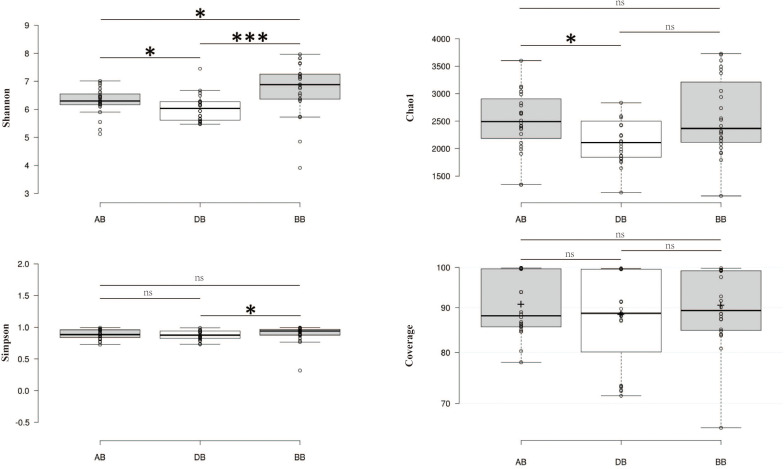
The α-diversity indices in different groups. The differences between pairs of two groups were tested by the Wilcoxon test. ns, not significant. ^∗^*p* < 0.05, ^∗∗∗^*p* < 0.001. BB, before *P. globosa* blooms; DB, during *P. globosa* blooms; AB, after *P. globosa* blooms.

After removing chimeras, a total of 135,841 OTUs were obtained from 72 samples, with 586–3796 (mean: 1887 ± 649) OTUs in each sample (Supplementary Table S2). In all samples (Figure 1), the bacterial profiles were dominated by Proteobacteria (68.2%), Bacteroidetes (15.5%), and Actinobacteria (12.1%). Good’s coverage for the samples, which provides an estimate of sampling completeness using a probability calculation with randomly selected sequences, was 89.91% (±8.7) on average when calculated with 97% similarity. This suggested that the majority of the bacterial phylotypes present in the samples were revealed. The Shannon index of BB was higher than those of AB and DB (Figure 2). The β-diversity of the surface bacterial community differed across the *P. globosa* blooms. The NMDS diagram showed divergence in β-diversity among the AB, DB, and BB groups (Supplementary Figure S2).

### Effects of *P. globosa* Density on the Phylogenetic Structure of the Water Surface Bacterial Community

The collected *P. globosa* colony diameter was 4 ± 1 mm. Various *P. globosa* densities were also observed at different bloom stages. In the DB groups, the estimated free-living cell density ranged from 1.26 × 10^3^ to 2.28 × 10^4^ cells/L, with a mean of 1.24 × 10^4^ cells/L. However, after *P. globosa* blooms, the density decreased to 7.91 ± 4.31 × 10^2^ cells/L throughout the bay (Supplementary Table S3). The two statistical metrics (mean NRI and mean NTI) were used to investigate the phylogenetic relatedness, which revealed whether deterministic or stochastic factors shape the species assemblages in marine surface water under various *P. globosa* levels (Table 1). The results presented similar patterns in terms of both the mean NRI and mean NTI. The mean NRI and mean NTI indicated low phylogenetic relatedness in AB, which suggested the dominance of stochastic factors in marine bacterial community assembly at the end of *P. globosa* blooms. Marine prokaryotes during *P. globosa* blooms showed higher phylogenetic relatedness (NRI > +2, *p* < 0.05; NTI > +2, *p* < 0.05) and thus the dominance of deterministic processes. The results also indicated that marine bacterial assembly was controlled by deterministic factors before *P. globosa* blooms in the study area (Table 1). However, the overall effect of *P. globosa* number did not show any significant correlations with the ecological processes (stochastic or deterministic) of the marine bacterial community (Table 1).

**TABLE 1:** Phylogenetic relatedness based on NRI and NTI of marine water samples.

Blooms period	Mean NRI ± SD	Null model percentile	Mean NTI ± SD	Null model percentile
BB	2.02 ± 1.16b	0.145	2.64 ± 0.58c	0.006
DB	2.33 ± 0.38a	0.016	2.53 ± 0.58c	0.002
AB	1.45 ± 0.37b	0.927	0.43 ± 0.28d	0.459

**Spearman‘s correlation**	**Coefficient Value**	***P*-value**	**Coefficient Value**	***P*-value**

Cell number of P. globasa	−0.22	0.24	0.18	0.46

*Values within a column followed by the same letter are not significantly different at P < 0.05.*

### Response Patterns of Marine Bacteria at the Genus Level to Environmental Variables in Different *P. globosa* Blooming Stages

**FIGURE 3:**
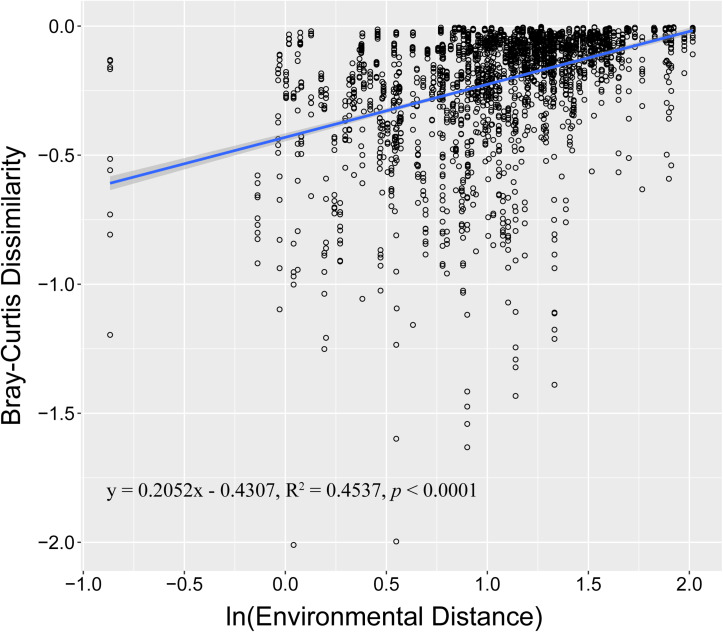
Regressions between environmental changes and microbial community similarity. The X-axis indicates the ln-transformed environmental distance (Euclidean), and the Y-axis indicates the ln-transformed Bray–Curtis distance. The solid blue line indicates the linear spline fit. The gray shadow represents the 95% confidence interval.

**FIGURE 4:**
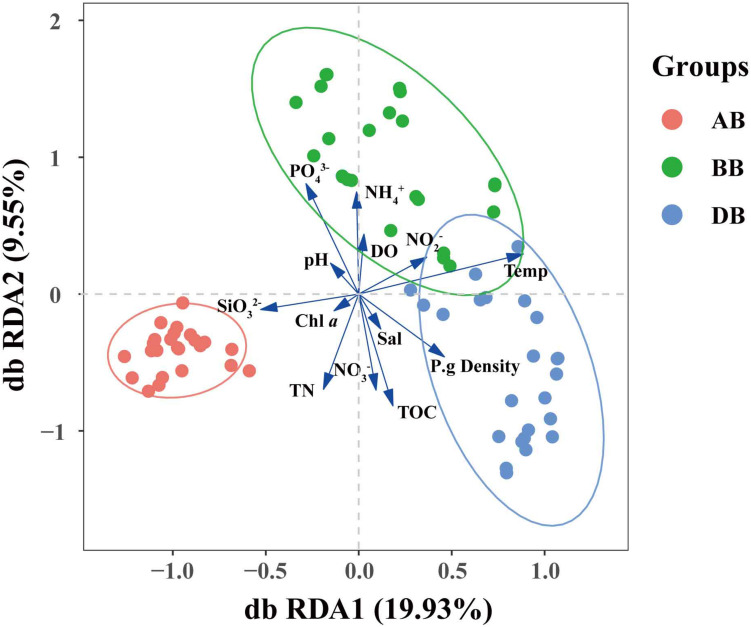
Distance-based redundancy analysis (db RDA) ordination plots of the bacterial community–environment relationships. BB, before *P. globosa* blooms; DB, during *P. globosa* blooms; AB, after *P. globosa* blooms.

During sample collection, temperature, salinity, pH, and dissolved oxygen (DO) were also measured. The temperature range was 12.38–16.98°C. The salinity range was 22.71–29.36 ppt (Supplementary Table S1). PERMANOVA revealed that environmental conditions significantly changed when *P. globosa* blooms occurred (*r*^2^ = 0.464, *p* < 0.001, Supplementary Table S1). However, environmental factors, such as pH, salinity, NO_2_^–^, and Chl *a*, were not significantly different among the different *P. globosa* bloom stages (ANOVA test, *p* > 0.05, Supplementary Table S1). For the total samples, regression analysis showed that community dissimilarity significantly increased with the degree of environmental variation (Figure 3). The relationships of the measured environmental parameters, including environmental factors (temperature, pH, salinity, DO, and Chl *a*), nutrients (PO_4_^3–^, NO_2_^–^, NO_3_^–^, NH_4_^+^, TN, SiO_3_^2–^, and TOC), and *P. globosa* density, with the bacterial communities analyzed (Figure 4). The results showed that the density of *P. globosa* was significantly connected with the variation in bacterial community structure (*r*^2^ = 0.3955, *p* < 0.001). Temperature (*r*^2^ = 0.8362, *p* < 0.001), PO_4_^3–^ (*r*^2^ = 0.6810, *p* < 0.001), and TOC (0.6535, *p* < 0.001) were the most influential factors affecting bacterial community structure.

**FIGURE 5:**
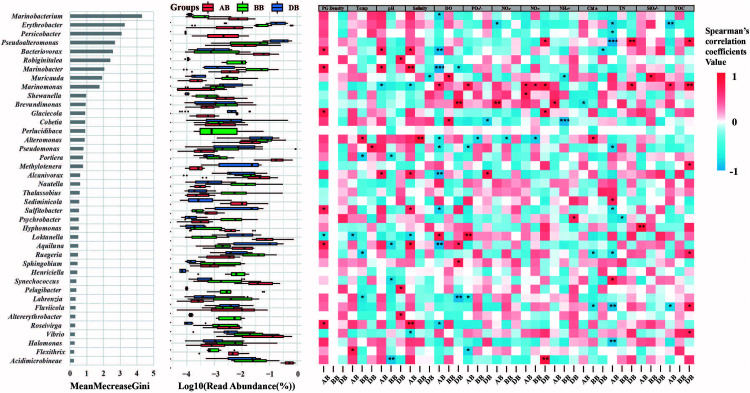
Top 40 most important genera for random forest classification. Left, the top 40 taxa were assessed by the Gini index, which indicated the importance of each genus in distinguishing various groups; middle, read abundances of the top 40 genera; right, the Spearman’s correlations between the relative abundances of the top 40 genera and the environmental parameters. PG density, *P. globosa* density; Temp, temperature; DO, dissolved oxygen; TN, total nitrogen; TOC, total organic carbon; BB, before *P. globosa* blooms; DB, during *P. globosa* blooms; AB, after *P. globosa* blooms. *p < 0.05, **p < 0.01, ***p < 0.001.

We also investigated the most important genera in different groups using the random forest method. The results showed that *Marinobacterium* (γ-proteobacteria), *Erythrobacter* (α-proteobacteria), *Persicobacter* (Cytophagales), *Pseudoalteromonas* (γ-proteobacteria), *Bacteriovorax* (γ-proteobacteria), and *Robiginitalea* (Flavobacteria) were the most important genera, with high Gini values (Figure 5), and the abundances of these genera were different among the AB, BB, and DB groups. Spearman’s correlation analysis showed that these genera had distinct relationships with *P. globosa* density in different *P. globosa* bloom stages (Figure 5). For example, *Marinobacterium* first presented a negative relationship with *P. globosa* density but then became positively correlated with it after the bloom. However, only seven species exhibited a significant positive or negative relationship with *P. globosa* density after the bloom (Figure 5). Moreover, the correlations between environmental parameters and the top 40 most abundant genera were also analyzed. The results showed that the surface free-living bacterial communities in the AB group were more correlated with environmental factors than were those in the BB and DB groups and played various roles in the abundances of these genera (Figure 5). For example, TN had significant relationships with 10 genera in the AB group but only two genera in the DB group.

### Factors Controlling Bacterial Community Composition During *P. globosa* Blooms

**FIGURE 6:**
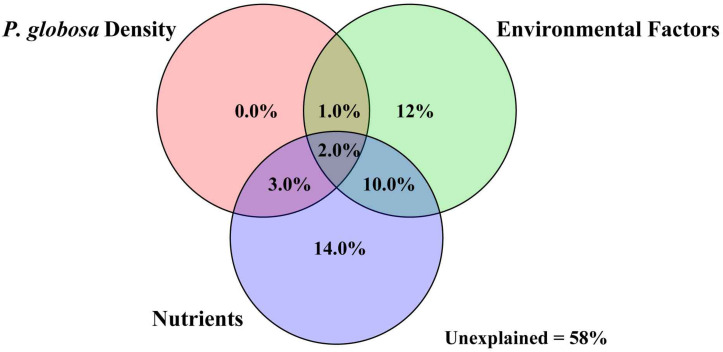
Variation partitioning analysis of *P. globosa* density, environmental factors, and nutrients against bacterial community structure.

**FIGURE 7:**
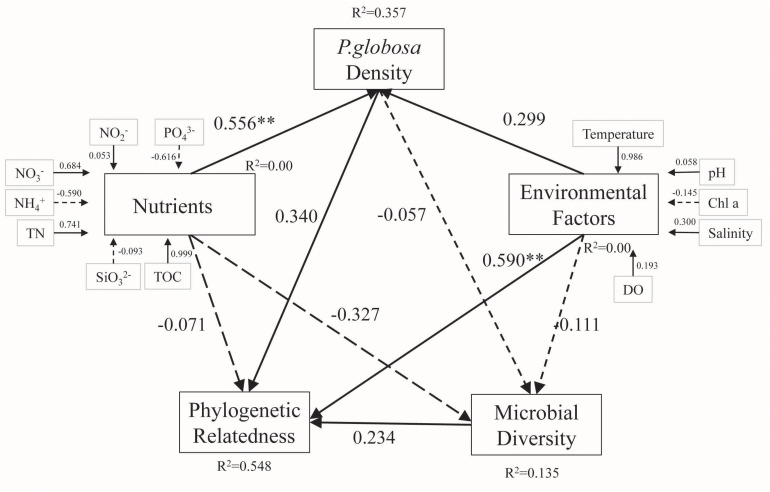
Path model based on the effects of environmental variables and relative abundance, diversity, and phylogenetic relatedness of the microbial community in marine waters with various *P. globosa* density levels. Solid and dotted lines indicate positive and negative effects, respectively. Asterisks indicate statistical significance (**p < 0.01).

VPA was performed to quantify the relative contributions of different environmental parameters to changes in bacterial community structure. *P. globosa* density, environmental factors, and nutrients explained 6, 25, and 27% of the observed variation, respectively, leaving 58% of the variation unexplained (one-way ANOVA, *p* < 0.01, Figure 6). To further reveal the possible pathways influencing free-living bacterial composition during *P. globosa* blooms, partial least squares (PLS) path modeling and multiple regression on distance matrices (MRM) analysis were implemented to identify the potential key drivers controlling bacterial structure. The modeling analysis provided the best fit to our data according to the respective indices of model fit (GOF = 0.73). The PLS analysis revealed that TN, NH_4_^+^, and NO_2_^–^ had a direct influence on *P. globosa* density, and *P. globosa* density influenced free-living bacterial phylogenetic relatedness both directly and indirectly through its effects on microbial diversity via nutrients and environmental factors. However, among these factors, only environmental variables (pH, Chl *a*, and salinity) explained significant fractions of the variance in bacterial assembly in the studied area (Figure 7).

## Discussion

### *P. globosa* Density Effects on Marine Bacterial Diversity and the Relative Abundances of Specific Taxa

Marine surface microbes are directly linked to ecosystem processes such as decomposition and biogeochemical cycles. The consequences of altered nutrient concentrations or altered phytoplankton communities may be reflected in changes in the ability to decompose or in the rate of degradation of organic materials. Therefore, it is important to explore the ecological processes of natural free-living microbial communities during environmental phytoplankton blooms. The prymnesiophyte *P. globosa* is one of the most globally distributed marine haptophytes, with the ability to generate high-biomass blooms (Riegman and Boekel, 1996; Schoemann et al., 2005; Zhang et al., 2015). As previous studies have shown, marine bacterial communities during *P. globosa* blooms in coastal waters are mainly dominated by Proteobacteria and Bacteroidetes, and bacterial structure can vary significantly during the bloom phase (Brussaard et al., 2005; Alison et al., 2014; Delmont et al., 2014; Wemheuer et al., 2015; Lars et al., 2017). Our data also revealed that the free-living bacterial communities during *P. globosa* blooms in the subtropical bay of the Beibu Gulf are mainly dominated by Proteobacteria and Bacteroidetes, and their compositions differ among groups. This finding suggests that *P. globosa* blooms may have similar impacts on the composition and diversity of marine bacteria. The bacterial community may also take advantage of the changing conditions during the *P. globosa* bloom.

Recent phylogenetic analyses of the marine microbiome have been carried out on both experimental and natural phytoplankton blooms, and the bacterial lineages of the most abundant bloom-associated microbes are now well-demonstrated (Brussaard et al., 2004, 2005; Alison et al., 2014; Bernd et al., 2015). For example, Roseobacter, flavobacteria, and members of α-Proteobacteria and γ-Proteobacteria are typically correlated with the successional patterns of the *P. globosa* population (Sheik et al., 2013b; Bernd et al., 2015). This evidence suggests that the application of bacterial bioindicators could systematically reflect and be used to record phytoplankton blooms. Random forest analysis is a good approach to investigate a highly relevant set of microbial biomarkers and for classification purposes (Dimucci et al., 2018). Our results indicated that *Bacteriovorax*, *Marinobacter*, *Glaciecola*, *Sulfitobacter*, *Lokanella*, *Aquiluna*, and *Roseivirga* were potential bacterial bioindicators for forecasting *P. globosa* blooms, with higher Gini values and significant correlations with *P. globosa* density after blooms (Figure 5). This result suggested that these genera have unique lineages associated with subtropical *P. globosa* blooms. For example, detection of a lower level of *Bacteriovorax* and/or other potential bacterial bioindicators may strongly indicate the end of a bloom. Further Spearman’s correlation analysis showed that the top 40 most abundant taxa were less influenced by *P. globosa* density. Phytoplankton blooms have a severe impact on bacterial communities, as they change nutrient availabilities and other environmental factors (Peperzak and Gabler-Schwarz, 2012). Thus, the results indicate that functional microbial diversity was altered not only by the *P. globosa* population but also by other sources of environmental variation.

NMDS analysis indicated that the β-diversity based on Bray–Curtis dissimilarity of free-living bacterial communities in marine waters in the AB group was distinct from that in the BB and DB groups (Supplementary Figure S2). Furthermore, the RDA revealed that PO_4_^3–^ was positively correlated with the BB group and negatively correlated with the AB and DB groups, while SiO_3_^2–^ had only a positive connection with the AB group (Figure 4). This suggests that *P. globosa* blooms can affect the bacterial community by environmental filtering of the various taxa in the blooms. Earlier studies suggested that peak abundances of phytoplankton coincided with low concentrations of DIN and DSi, while high abundances of dinoflagellates coincided with low values of DIP (Wang et al., 2006). In laboratory studies, it has been suggested that the decay of *P. globosa* blooms results in phosphate limitation and N-deficient conditions (Brussaard et al., 2005; Bo et al., 2015). Therefore, this result supports the view that environmental conditions determine which species are present during *P. globosa* blooms. Interestingly, in this study, we did not detect the expected shifts in the Chl *a* and nitrate profiles following changes in *P. globosa* abundance across the different sampling situations. This is mostly true during *P. globosa* blooms, which impact the food web structures in the environment, particularly through a negative impact on co-occurring phytoplankton through allelochemical and toxic effects (Veldhuis and Wassmann, 2005). This may not cause a significantly different concentration of Chl *a*. Because NO_3_^–^ is related to specific protein synthesis for *P. globosa* colonies (Xiaodong et al., 2011). NO_3_^–^ is expected to be consumed during the bloom. However, the values were higher during the bloom than before the bloom. This suggests that *P. globosa* is not the main contributor to nitrate unitization in the local ecosystem. Therefore, it is important to carry out nutrient uptake kinetics experiments to evaluate the specific roles of *P. globosa* in marine elemental cycles.

Moreover, PLS path modeling and multiple regression of *P. globosa* density, nutrients, and environmental factors were implemented to further reveal the possible causal pathways influencing marine free-living microbial diversity and phylogenetic relatedness during *P. globosa* blooms. Nutrients, such as TOC (path coefficient = 0.999), TN (path coefficient = 0.741), NO_2_^–^ (path coefficient = 0.684), and PO_4_^3–^ (path coefficient = 0.616), and temperature (path coefficient = 0.986) influenced *P. globosa* density directly or indirectly. These findings are consistent with current knowledge of the physiology of *P. globosa*; for example, as a temperature-dependent marine phytoplankton (Schoemann et al., 2005; Bernd et al., 2015), this species has the ability to utilize both organic phosphorus/phosphate and organic carbon and is competitive at high nitrate levels (Stefels, 2000; Tungaraza et al., 2003; Blauw et al., 2010; Sheik et al., 2013b). Nitrogen and TOC are generally agreed to be key factors affecting variation in microbial diversity and phylogenetic relatedness because they have been extensively reported as major determinants of microbial communities in global marine ecosystems. Based on PLS-LM analysis, in this study, we also found that environmental factors (path coefficient = 0.590, *p* < 0.05) had greater direct effects on phylogenetic community composition than *P. globosa* (path coefficient = 0.590) and microbial diversity (path coefficient = 0.234), while nutrients (path coefficient = -0.071) had a weak direct influence. These results emphasize the significant role of PO_4_^3–^, TN, and TOC concentrations and temperature variation in determining the abundance of and variation in the bacterial community, and *P. globosa* density may not be a key driver of marine free-living bacterial assembly.

### *P. globosa* Density Effects on Species Assembly of the Microbial Community

Although there is an extensive literature examining microbial community composition and gene expression patterns during phytoplankton blooms in marine environments (Zubkov et al., 2001; Lamy et al., 2009; Loureiro et al., 2011; Sheik et al., 2013b), few studies have examined how the relative influences of stochastic and deterministic processes change with environmental conditions. We speculated that deterministic processes shape bacterial community structure through environmental filtering effects at every stage of *P. globosa* blooms. The results for the mean NRI and NTI showed a greater effect of deterministic factors on the community assembly of marine microbiomes in the non-bloom and blooming stages. However, our results also indicated that there is greater importance of stochastic factors in marine waters at the end stage of *P. globosa* blooms. The relative importance of stochastic factors possibly depends on unpredictable short-term pulse disturbance. Recent studies confirmed that during the terminal stage of the bloom, *P. globosa* can produce extremely large amounts of anti-metabolites and dissolved organic carbon to disrupt intimate relationships of bacterial lineages with the *P. globosa* population (Peperzak and Gabler-Schwarz, 2012; Alison et al., 2014). Meanwhile, some bacteria, such as flavobacteria and other γ-proteobacteria, produce algicidal compounds that shift the interaction from mutualistic to pathogenic. Previous studies (Zhou et al., 2014; Ren et al., 2017) also indicated that an increase in nutrients in water may disrupt the metabolic kinetics and growth rate of the bacterial community, leading to random colonization or extinction, unpredictable perturbations, and amplification of the initial differences in bacterial composition. Therefore, the influence of stochastic processes increased with these uncertain factors, disrupting the metabolic kinetics and growth rates of abundant and rare species at the end stage of the blooms.

Our results confirmed the simultaneous effects of stochastic and deterministic drivers on bacterial community assembly during *P. globosa* blooms. However, significant correlations were not observed between the mean NRI/NTI and *P. globosa* density. Although most measured environmental variations showed no significant correlation with NTI, temperature, salinity, NO_2_^–^, or SiO_3_^2–^, the mean NRI values were found to be more sensitive to environmental variation. These results indicated that deterministic selection may be caused by specific undetected environmental factors. Our results based on path modeling revealed that increases in *P. globosa* density, environmental factors (pH, salinity, and temperature), and microbial diversity will positively affect phylogenetic relatedness, thus explaining the dominance of deterministic processes. Early studies demonstrated that *P. globosa* plays a key role as an intermediary in the transfer of elements in marine ecosystems, such as the release of organic material upon *P. globosa* lysis, which plays a key role in structuring bacterial communities and affecting the cycling of biolimiting elements (Peperzak and Gabler-Schwarz, 2012; Sheik et al., 2013a; Speeckaert et al., 2018). Therefore, our results suggested that the assembly of the marine free-living bacterial community during *P. globosa* blooms is a complex process with mixed effects exerted by the *P. globosa* population, marine microbiomes, and environmental parameters. *P. globosa* may involve microbial community assembly processes as a key regulator by stimulating functional genera to drive marine bacterial community turnover.

The most striking feature of *P. globosa* is that it can form gel-like colonies ranging from several millimeters to centimeters in diameter (Verity and Medlin, 2003), which can produce viscous, slimy, and springy brown jelly layers, thus modifying the rheological properties of seawater. Past studies have shown that a majority of aquatic bacteria pass through 0.45 μm filters (Hahn, 2004; Whipple et al., 2005; Ghuneim et al., 2018). Therefore, we used 0.8 and 0.45 μm prefilters to eliminate the majority of debris and particles and removed the remaining humic and other non-organic materials, which led to the loss of information about the microbial communities attached to larger organisms and to *P. globosa* colonies. However, certain larger bacteria were also abundant in the samples, such as *Nocardioides marinus*, which is approximately 1.0–1.8 μm long and 0.4–0.6 μm wide (Choi et al., 2007). This may be due to the finding of larger bacteria in their starvation forms, which allowed them to pass through the filters (Ghuneim et al., 2018). Nevertheless, future research on related topics should consider non-free-living bacterial communities by detecting every size fraction of DNA and may emphasize the interactive effects of various environmental changes on microbial community assembly under *P. globosa* blooms in laboratory experiments and environmental tests.

## Conclusion

This study revealed the interactive effects of both *P. globosa* density and environmental parameters on the marine free-living bacterial community. Both *P. globosa* density and environmental variations affected α-diversity and significantly change species turnover. The bacterial community in marine water at the non-blooming and blooming stages tended to be controlled by deterministic factors, while the bacteria in marine waters at the terminal stage of the blooming were controlled by stochastic factors. In addition, several key taxa strongly associated with the *P. globosa* population. Overall, our results suggested that various communities may show similar functional responses driven more by the *P. globosa* population and environmental parameters than by community structure.

## Data Availability Statement

The datasets generated for this study were deposited in NCBI Sequence Read Archive (SRA) under BioProject number PRJNA565408.

## Author Contributions

NL and ZK prepared the manuscript. HZ, GJ, and QX analyzed the data. JT, XL, JW, HL, and CT prepared the sampling and treating the samples. All authors contributed to the article and approved the submitted version.

## Conflict of Interest

The authors declare that the research was conducted in the absence of any commercial or financial relationships that could be construed as a potential conflict of interest.

## Footnotes

1 https://mothur.org/wiki/miseq_sop/
