# Supplementary material for: Phylogenetic Responses of Marine Free-Living Bacterial Community to Phaeocystis globosa Bloom in Beibu Gulf, China
Source: Front Microbiol. 2020 Jul 16;11:1624. doi: 10.3389/fmicb.2020.01624 (PMC7378386; doi:10.3389/fmicb.2020.01624)
Supplement: Supplementary file 1 [file Data_Sheet_1.DOCX]

**Table S1 Environmental factors of the Phaeocystis project in Qinzhou Bay, 2017-2018**

**Table S2 Sequencing results and α-diversity index values of the marine water samples**

**AB, after P. golobosa blooms; BB, before P. golobosa blooms; DB, during P. golobosa blooms**.

**Table S3 Density of *P. globosa* at each water samples**

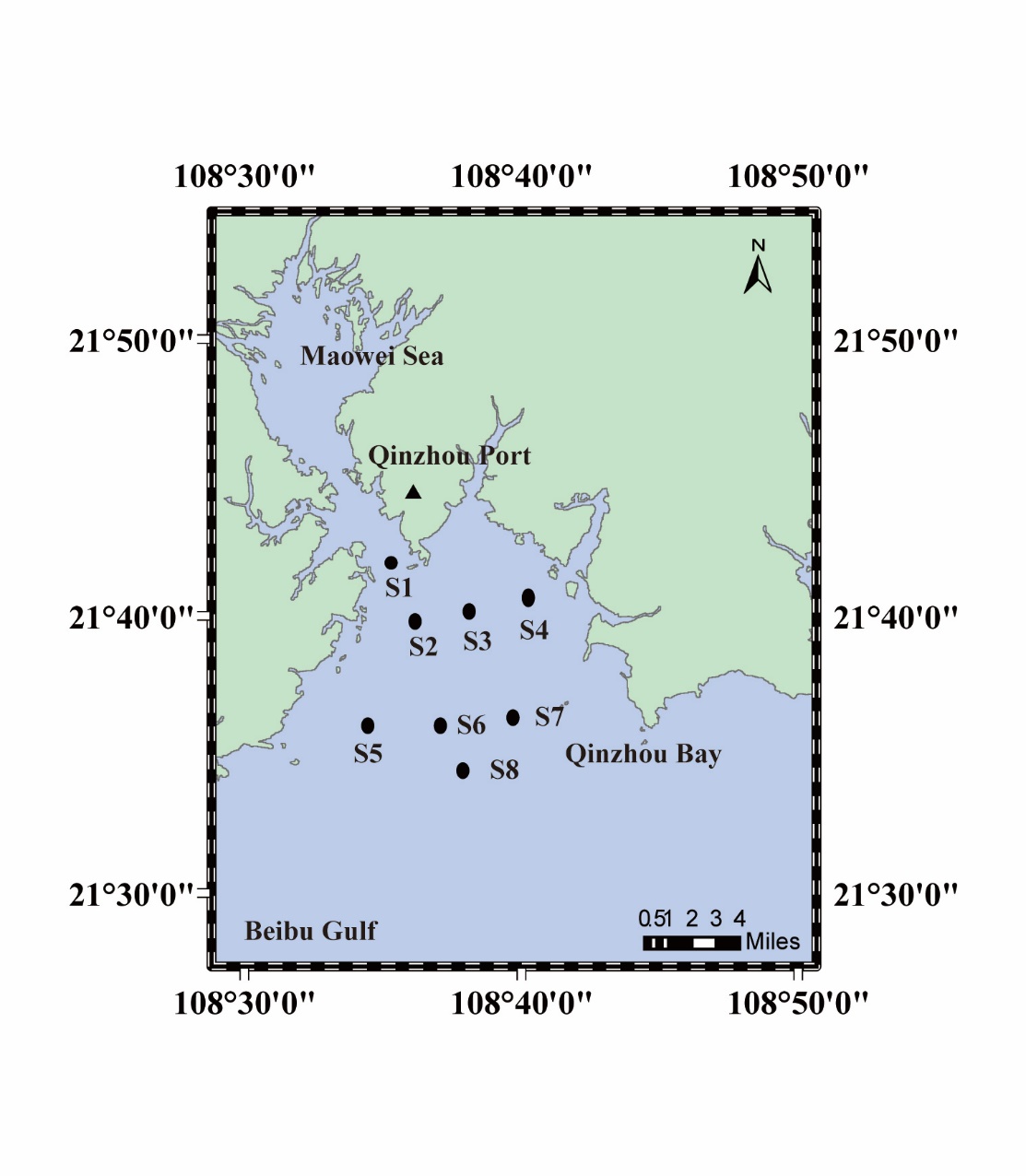


**Figure S1 Sampling sites of the marine waters in Beibu Gulf in China**

**
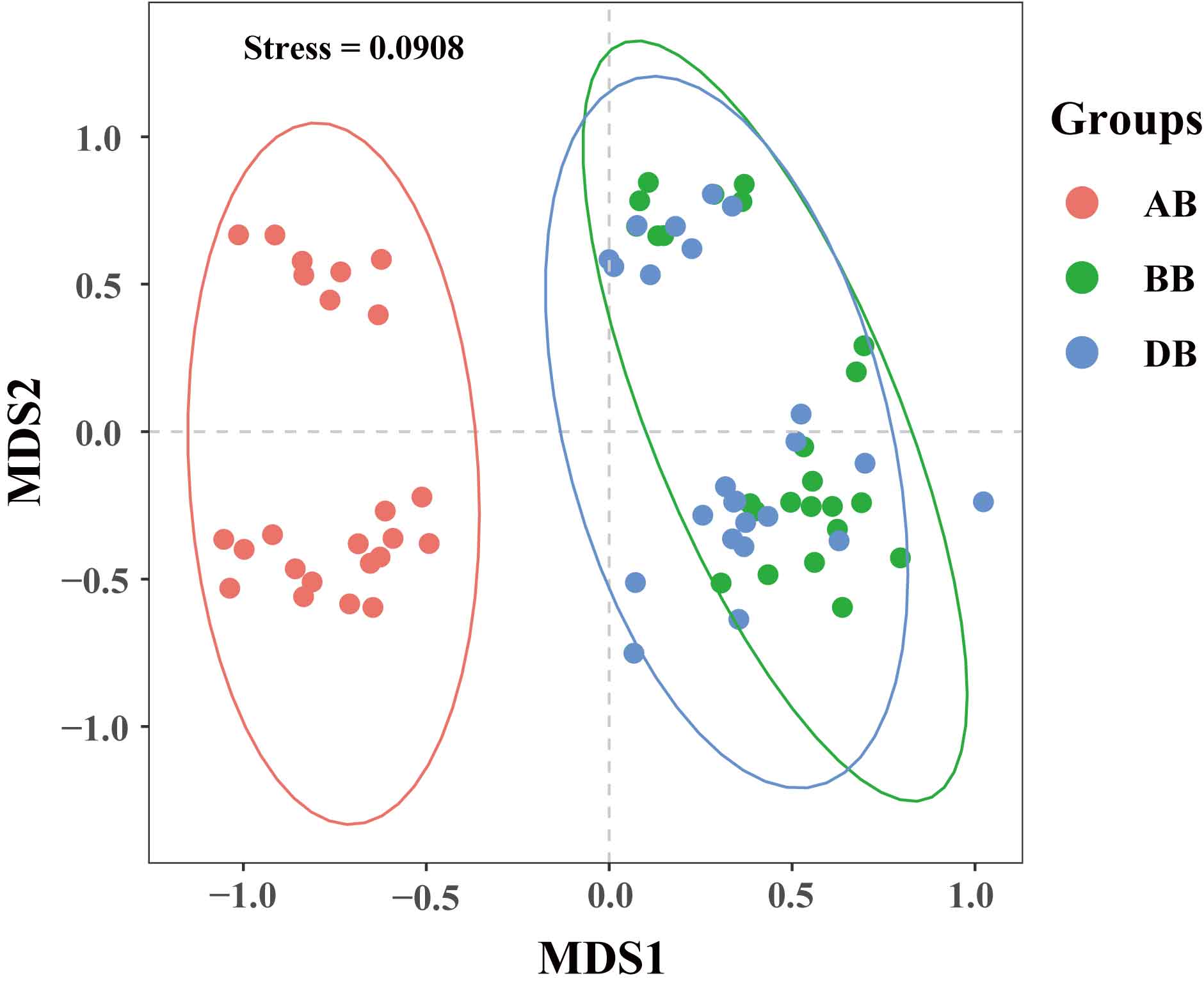
**

**Figure S2 Non-metric multidimensional scaling ordination (NMDS) plot showing β-diversity pattern based on Bray-Curtis dissimilarity of bacterial community in marine waters.**
